# Deciphering a mitochondria-related signature to supervise prognosis and immunotherapy in hepatocellular carcinoma

**DOI:** 10.3389/fimmu.2022.1070593

**Published:** 2022-12-02

**Authors:** Yanlong Shi, Guo Huang, Fei Jiang, Jun Zhu, Qiyang Xu, Hanlu Fang, Sheng Lan, Ziyuan Pan, Haokun Jian, Li Li, Yewei Zhang

**Affiliations:** ^1^ Hepatopancreatobiliary Center, The Second Affiliated Hospital of Nanjing Medical University, Nanjing, Jiangsu, China; ^2^ Hengyang Medical School, University of South China, Hengyang, Hunan, China; ^3^ Key Laboratory of Tumor Cellular and Molecular Pathology, College of Hunan Province, Cancer Research Institute, University of South China, Hengyang, Hunan, China; ^4^ Department of General Surgery, Fuyang Hospital of Anhui Medical University, Fuyang, Anhui, China; ^5^ Department of Oncology, Fuyang Hospital of Anhui Medical University, Fuyang, Anhui, China; ^6^ Department of General Surgery, the Fifth People’s Hospital of Fuyang City, Fuyang, Anhui, China; ^7^ Institute of Medical and Health Science, Hebei Medical University, Shijiazhuang, Hebei, China; ^8^ The Second Clinical College of Guangzhou Medical University, Guangzhou, Guangdong, China; ^9^ Hengyang Hospital affiliated of Hunan University of Chinese Medicine, Hengyang, Hunan, China; ^10^ School of Basic Medical Sciences, Xinxiang Medical University, Xinxiang, Henan, China

**Keywords:** mitochondria, hepatocellular carcinoma, consensus clustering, tumor microenvironment, immunotherapy, drug sensitivity

## Abstract

**Background:**

Hepatocellular carcinoma (HCC) is a major public health problem in humans. The imbalance of mitochondrial function has been discovered to be closely related to the development of cancer recently. However, the role of mitochondrial-related genes in HCC remains unclear.

**Methods:**

The RNA-sequencing profiles and patient information of 365 samples were derived from the Cancer Genome Atlas (TCGA) dataset. The mitochondria-related prognostic model was established by univariate Cox regression analysis and LASSO Cox regression analysis. We further determined the differences in immunity and drug sensitivity between low- and high-risk groups. Validation data were obtained from the International Cancer Genome Consortium (ICGC) dataset of patients with HCC. The protein and mRNA expression of six mitochondria-related genes in tissues and cell lines was verified by immunohistochemistry and qRT-PCR.

**Results:**

The six mitochondria-related gene signature was constructed for better prognosis forecasting and immunity, based on which patients were divided into high-risk and low-risk groups. The ROC curve, nomogram, and calibration curve exhibited admirable clinical predictive performance of the model. The risk score was associated with clinicopathological characteristics and proved to be an independent prognostic factor in patients with HCC. The above results were verified in the ICGC validation cohort. Compared with normal tissues and cell lines, the protein and mRNA expression of six mitochondria-related genes was upregulated in HCC tissues and cell lines.

**Conclusion:**

The signature could be an independent factor that supervises the immunotherapy response of HCC patients and possess vital guidance value for clinical diagnosis and treatment.

## Introduction

Hepatocellular carcinoma (HCC) is the second cause of cancer-related death, characterized by an advanced stage at the first diagnosis, threatening public health security and human happiness (1). Genetic and epigenetic alterations give rise to extensive tumor heterogeneity, which results in an unsatisfactory prognosis and a heavy disease burden for HCC patients (2, 3). Moreover, the 5-year survival rate of HCC patients is about 40% after radical hepatectomy (4). Currently, the prediction of HCC patients’ survival primarily depends on the clinical-pathological staging system, such as the Barcelona-Clinic Liver Cancer staging system (5, 6). However, patients with the same pathological stage may have different prognoses (7). Given the above, there is an urgent need for a novel signature to determine the prognosis of patients with HCC and to further comprehend the underlying mechanism of progression.

Mitochondria are the power stations in eukaryotic cells, which are responsible for information transmission, cell differentiation, and apoptosis by integrating metabolic pathways such as oxidative phosphorylation and participating in cell metabolism, proliferation, and programmed cell death (8, 9). Mitochondrial dysfunction has a crucial influence on the biological function of cells and leads to the occurrence of diseases (10). Moreover, epigenetic regulation of mitochondrial-related genes plays a vital role in the progression and treatment of cancers (11, 12). Studies reported that defects in mitochondrial-related genes regulate the immune metabolism microenvironment by promoting glycolysis and lactic acid synthesis in HCC (13). However, the prognosis and immune value of mitochondrial-related genes in HCC have not been reported.

Herein, we constructed a novel mitochondrial-related signature in HCC to identify its potential relationship with prognosis, cell functions, immunity, and drug sensitivity based on risk groups and risk scores. Subsequently, we further verified the expression of six mitochondrial-related genes in HCC tissues and cell lines by experiments. This study aims to determine the prognosis of HCC patients through using the mitochondrial-related signature and to demonstrate the possible molecular mechanisms in HCC.

## Methods

### Data acquisition

The 365 HCC patients whose RNA sequencing data and relevant clinical information were obtained from The Cancer Genome Atlas (TCGA) database are regarded as the training cohort. The expression and information of 231 HCC patients were downloaded from the ICGC database as a validation cohort.

### Identification of prognostic mitochondria-related genes

As presented in **Supplementary Table 1**, the 1,136 mitochondria-related genes were extracted from MitoCarta3.0 (14). The differentially expressed genes (DEGs) were conducted by the “limma” R package in HCC and the relevant normal tissues, with the threshold of |log_2_FC| >2 and FDR <0.05. The “VennDiagram” R package was used to identify prognostic mitochondria-related genes.

### Development of mitochondria-based prognostic model

Univariate Cox regression was used to explore the potential prognostic mitochondria-related genes in HCC patients and the hazard ratio (HR) was shown. The mitochondria-based prognostic model was established by Lasso Cox regression and the R package “glmnet,” and 10-fold cross validation was employed to test the feasibility of the signature (15). The independent and dependent variables were candidate genes for the prognostic model and HCC patients’ survival times, respectively. According to the optimum parameter, the risk score was derived from the expression of candidate genes with standardized regression coefficients. The concrete formula is risk score = (MTHFD1L expression ∗ 0.109) + (NT5DC2 expression ∗ 0.156) + (POLQ expression ∗ 0.385) + (RECQL4 expression ∗ 0.017) + (TOMM40L expression ∗ 0.243) + (TXNRD1 expression ∗ 0.221). Then, HCC patients were classified into low- and high-risk groups because of the median risk score. Subsequently, the R packages “Rtsne” and “ggplot2” were used to examine the characteristics of principal component analysis (PCA) and t-distributed random neighbor embedding (t-SNE). The prognostic value of the model was estimated by the R packages “survminer” and “timeROC.” In addition, the predictive probability of the mitochondria-based prognostic model was observed from nomogram, which established a risk score and clinicopathological features using the R packages “rms” and “regplot.” The calibration curves and C-index value were introduced to verify the accuracy of the nomogram and risk score.

### Prognostic value analysis of risk score

Multivariate Cox regression was investigated to judge the model’s independent prognostic value and the relationship between the risk score and clinical features (age, gender, grade, and tumor stage) in the TCGA and ICGC cohorts, and the visualized results were presented by the R package “forestplot.”

### Functional enrichment analysis based on risk groups

The R package “ClusterProfiler” was determined to the functional enrichment including Gene Ontology (GO) and Kyoto Encyclopedia of Genes and Genomes (KEGG) between the low- and high-risk groups, with the threshold of Mann–Whitney test, |log2FC| ≥1, and FDR <0.05.

### Evaluation of tumor microenvironment, immune response, and immune subtypes

To identify the expression of immune cells and immune functions in low- and high-risk groups, we further conducted using the R package “GSVA” (16). The composition of 22 tumors associated with immune infiltrate cells was investigated by the R package “CIBERSORT” in risk groups. Spearman’s regression analyzed the infiltration of immune cells for the stromal score and immune score. The relationship between tumor stem cells and risk scores was evaluated by Spearman’s correlation. Moreover, we explored immune checkpoints and immune subtypes in low- and high-risk groups using the R package “ggpubr” and were tested by one-way ANOVA analysis.

### Investigation of the prognosis and immune characteristics based on clusters

According to the consensus cluster analysis, the R package “ConsensusClusterPlus” was used to estimate prognosis and immune characteristics in HCC patients (17). The R packages “Rtsne” and “ggplot2” were used to compare the characteristics of PCA and t-SNE among clusters. The Kaplan–Meier curve in the “survival” R package visualizes survival times in different clusters. The ESTIMATE score, immune score, and stromal score were obtained from each cluster’s R package “ESTIMATE.” In the TIMER database, we analyzed immune cell infiltration in HCC patient clusters based on various algorithms.

### Tissue samples

We collected 10 pairs of HCC and adjacent non-tumor tissues from Fuyang Hospital of Anhui Medical University, which were authorized by HCC patients and the Ethics Committee of the Hospital.

### Cell culture

The human HCC cell lines (7721 and HepG2) and hepatic normal cells (LO2) were donated by the First Hospital Affiliated to Anhui Medical University. The cell culture conditions were as follows: DMEM with high glucose (HyClone), 10% fetal bovine serum (VivaCell, Shanghai, China), 5% CO_2_, and 37°C.

### Quantitative real-time polymerase chain reaction

The total RNA of tissues and cells was extracted using the TRIzol reagent (Takara). According to the instructions, reverse transcription was applied to the PrimeScript™ kit (Takara). Subsequently, the SYBR Green qPCR Mix was used to detect the relative expression of signature genes using the 2^−ΔΔCt^ method. The primer sequences used for the experiment are listed in **Supplementary Table 2**.

### Analysis of immunohistochemistry

Immunohistochemistry was used to detect the protein expression of six mitochondria-related genes. We analyzed the images of five-gene protein expression in “tissue” and “pathology” of modules in the HPA database. Patients and image serial numbers are included in this study. All images were re-judged by two pathologists. The regents and citations are as follows: MTHFD1L: Atlas Antibodies Cat#HPA029041, RRID: AB_2672880, dilution: 1:350; NT5DC2: Atlas Antibodies Cat#HPA050683, RRID: AB_2681215, dilution: 1:40; RECQL4: Atlas Antibodies Cat#HPA025821, RRID: AB_1856168, dilution: 1:40; TOMM40L: Atlas Antibodies Cat#HPA051304, RRID: AB_2681432, dilution: 1:75; TXNRD1: Santa Cruz Biotechnology Cat#sc-28321, RRID: AB_628405, dilution: 1:2500; POLQ: Atlas Antibodies Cat#HPA026762, RRID: AB_10601248, dilution: 1:450.

### Statistical analysis

The one-way analysis of variance in HCC and normal tissues examined the DEGs. Cox regression with hazard ratios (HRs) and 95% confidence intervals (CIs) was employed to estimate prognosis. Those experimental results were processed using one-way ANOVA analysis using GraphPad Prism 8.0 software. The R software (version 4.0) was applied to statistical analyses, with p <0.05 considered significant.

## Results

### Identification of prognostic mitochondria-related genes

**Figure 1:**
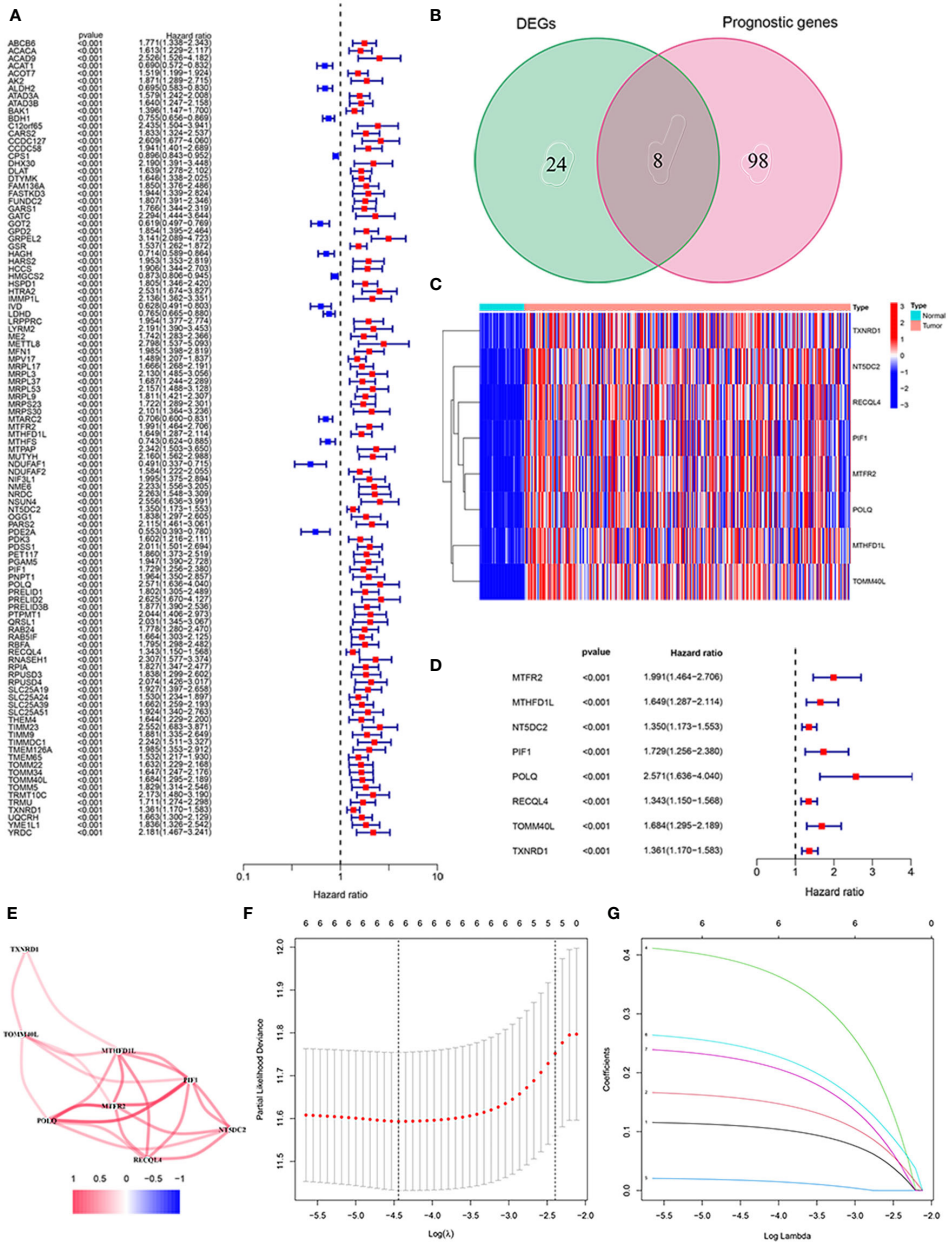
Identification of the mitochondria-related prognostic signature in the TCGA cohort. **(A)** The association between prognostic gene expression and OS by forest plot. **(B)** Venn diagram to classify the DEG between HCC and adjacent normal tissues. **(C)** Heat map of the expression of eight overlapping genes. **(D)** Univariate Cox regression analysis of eight overlapping genes related to OS. **(E)** Correlation network of prognostic genes. **(F)** LASSO regression with tenfold cross-validation identified ten prognostic genes using the minimum λ. **(G)** LASSO coefficient profiles of six prognostic genes in HCC.

As shown in **Figure 1A**, there were 106 prognostic genes differentially expressed in HCC and the corresponding normal tissues. Combined with 32 DEGs related to mitochondria in HCC, we screened eight mitochondria-related genes by Venn diagram (**Figure 1B**). The heatmap analysis suggested that the eight mitochondria-related genes were up-regulated in HCC tissues compared to normal tissues (**Figure 1C**). We identified the eight mitochondria-related genes as prognostic factors, which are regarded as hazards with a risk ratio greater than 1 (**Figure 1D**). **Figure 1E** presents the correlation among these genes.

### Construction and validation of a novel mitochondria-related gene signature

**Figure 2:**
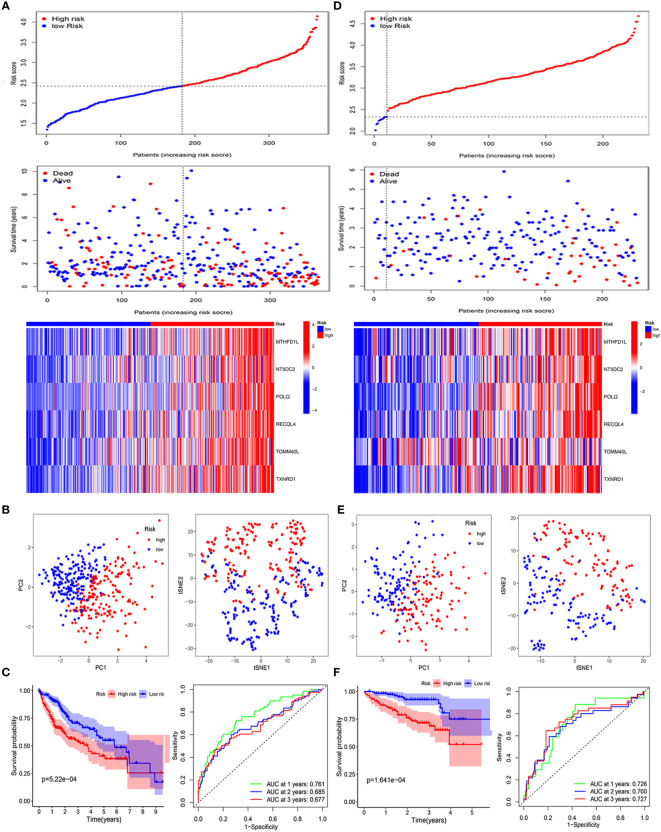
Validation of the six mitochondria-related prognostic signatures in the TCGA and ICGC cohorts. TCGA cohort: **(A–C)**; ICGC cohort: **(D–F)**. **(A–D)** Risk score values and distribution, OS status and heatmap analysis of the 6-gene signature model. **(B, E)** The PCA plots and t-SNE analysis of risk scores. **(C, F)** Kaplan–Meier curves and AUC time-dependent ROC curves for OS.

We constructed a prognostic signature of six mitochondria-related genes using LASSO COX regression analysis, which was judged by optimum λ values. The risk score = 0.109 ∗ (MTHFD1L expression) + 0.156 ∗ (NT5DC2 expression) + 0.385 ∗ (POLQ expression) + 0.017 ∗ (RECQL4 expression) + 0.243 ∗ (TOMM40L expression) + 0.221 ∗ (TXNRD1 expression) (**Figures 1F** **, G**). In **Table 1**, we suggested that the risk score was related to gender and tumor grade in the high-risk group of HCC patients. According to median risk scores, the patients with HCC were divided into low- and high-risk groups in the TCGA cohort (**Figure 2A**). As shown in **Figure 2B**, the PCA and t-SNE analyses, there are obvious clusters between low- and high-risk groups. The results of patients in the high-risk group show a higher risk score and shorter survival time (**Figure 2C**). The ROC curves of the 1-year, 2-year, and 3-year OS rates demonstrated the sensitivity and specificity of the model with values of 0.761, 0.685, and 0.677, respectively. Furthermore, the Kaplan–Meier analysis suggested the two groups was a statistical difference in survival in the TCGA cohort (P = 5.22e−04). The expression of MTHFD1L, NT5DC2, POLQ, RECQL4, TOMM40L, and TXNRD1 in the low-expression group had a longer survival time (**Supplementary Figure 1**). In addition, 151 HCC patients were referred to as the validation set in the ICGC cohort. Based on the same median risk score as the TCGA cohort, the ICGC cohort was classified into low- and high-risk groups with 11 and 220 HCC patients, respectively (**Figure 2D**). The PCA and SNE analyses reconfirmed the difference in distribution between the two groups (**Figure 2E**). Moreover, compared with high-risk group, patients in low-risk groups had a better OS (P = 1.641e−04). The AUC value of the ROC analysis was 0.726 at 1-year, 0.700 at 2-year, and 0.727 at 3-year survival (**Figure 2F**).

**Table 1:** The association of clinicopathological features.

Characteristics	TCGA-LIHC cohort			ICGC-LIHC cohort		
	High risk (%)	Low risk(%)	*P* value	High risk(%)	Low risk(%)	*P* value
**Age**						
≤65 year	115(63.19)	112(61.20)	0.777	57(25.91)	4(36.36)	0.677
>65 year	67(36.81)	71(38.80)		163(74.09)	7(63.64)	
**Gender**						
FEMALE	50(27.47)	69(37.70)	0.048	86(39.09)	3(27.27)	0.639
MALE	132(72.53)	114(62.30)		134(60.91)	8(72.73)	
**Grade**						
G1-2	102(56.04)	128(69.95)	0.006			
G3-4	78(42.86)	52(28.42)				
unknow	2(1.1)	3(1.64)				
**Stage**						
I-II	124(68.13)	130(71.04)	0.475	132(60)	9(81.82)	0.258
III-IV	47(25.82)	40(21.86)		88(40)	2(18.18)	
unknow	11(6.04)	13(7.1)		0	0	

### Prognostic value of mitochondria-related gene model

**Figure 3:**
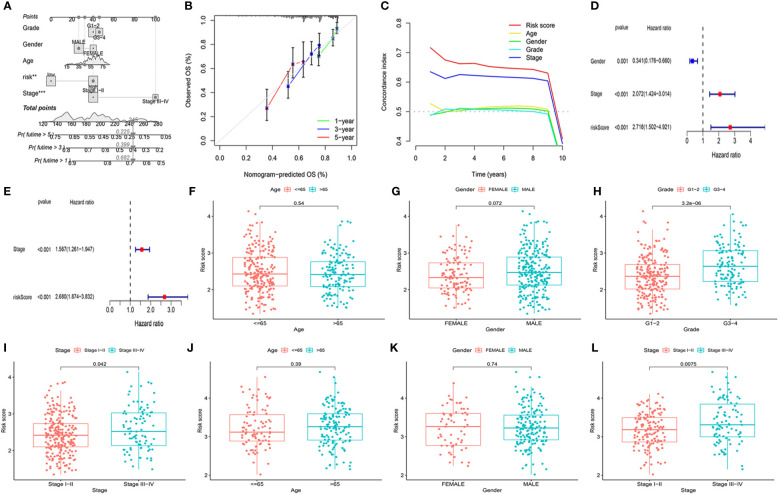
Evaluation of the prognostic signature and association of the risk score with clinicopathological characteristics. **(A)** Construction of a nomogram to predict the 1-year, 3-year, and 5-year survival probabilities based on OS-related pathological features. **(B)** The calibration curve was to verify the efficacy of nomogram. **(C)** Evaluation the superiority of the risk score by the C-index. **(D, E)** Screening of OS-related pathological features by multivariate Cox regression in the TCGA and ICGC cohorts. TCGA cohort: **(F)** Age. **(G)** Gender. **(H)** Tumor grade. **(I)** Tumor stage. ICGC cohort: **(J)** Age. **(K)** Gender. **(L)** Tumor stage. **P<0.01, ***P<0.001.

To better evaluate the prognosis of HCC patients, we developed a Nomo diagram, including mitochondria-related gene signatures, grade, age, gender, risk score, and stage, to forecast the survival probability at 1, 3, and 5 years (**Figure 3A**). In **Figure 3B**, the OS predicted by nomogram was in good agreement with those observed OS in calibration curves. Moreover, the C-index demonstrated the prognostic model possessed greater superiority than other characteristics (**Figure 3C**).

To identify whether the mitochondria-related gene model acted as an independent prognostic indicator, we further investigated it using the multivariable Cox regression analysis. **Figures 3D** **, E** indicated that the risk score was observably related to OS in the TCGA cohort (P <0.001, HR = 2.718 (1.502–4.921)) and ICGC cohort (P <0.001, HR = 2.680 (1.874–3.832)). Combined with clinicopathological characteristics, there were significantly different tumor stages I–II and tumor stage III–IV in patients in the TCGA cohort (P = 0.042) and the ICGC cohort (P = 0.008) (**Figures 3F** **–L**). According to the TCGA cohort, the risk score was also correlated with tumor grade (P = 3.2e−06). The above results demonstrated that the mitochondria-based six-gene model could be an adverse prognostic model for patients with HCC.

### Assessment of immune status and tumor microenvironment

**Figure 4:**
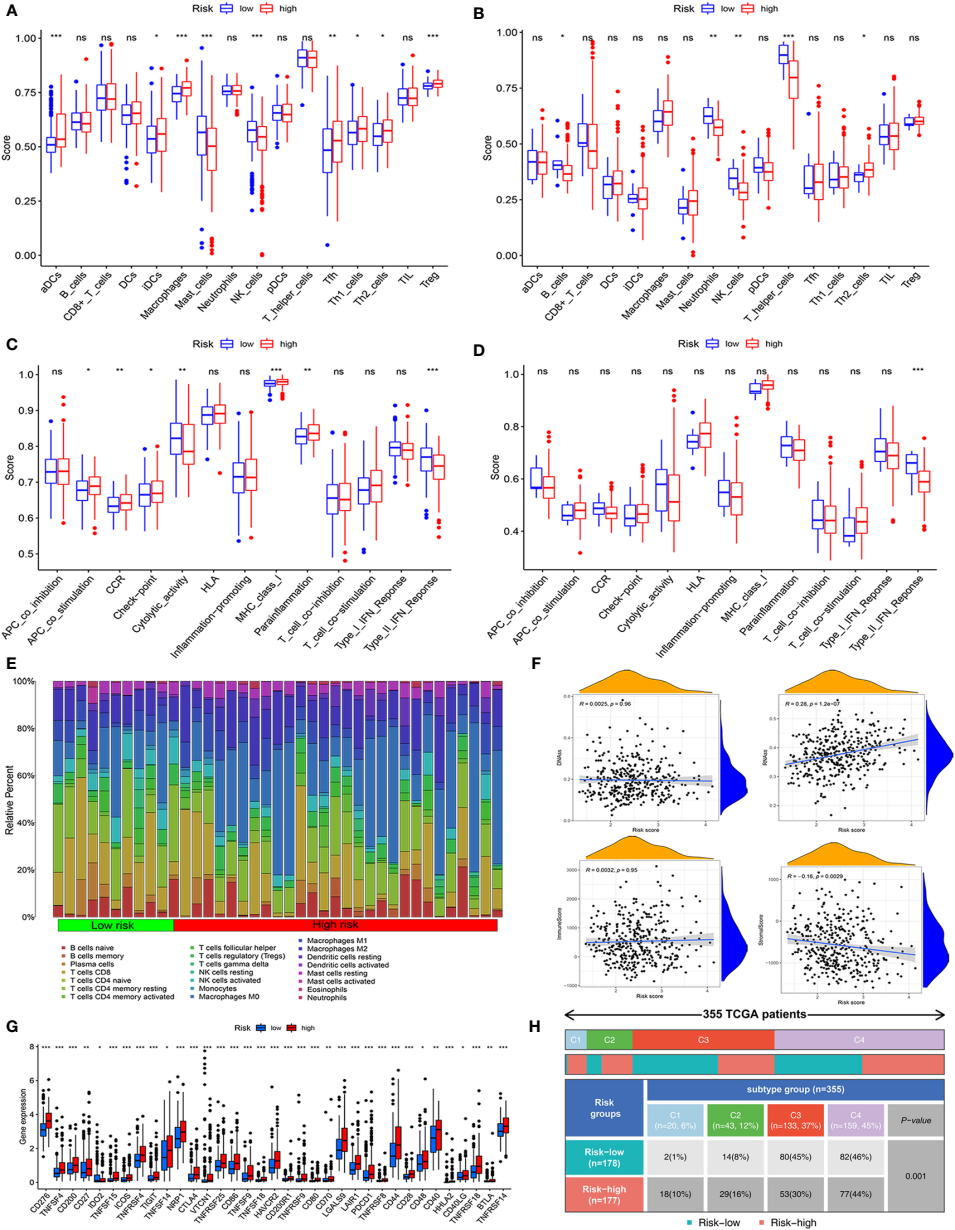
Assessment of immune status, tumor microenvironment, and immune checkpoints of mitochondria-related prognostic signatures. **(A, B)** The scores of 16 immune cells were detected by ssGSEA analysis based on low- and high-risk groups in the TCGA cohort and the ICGC cohort.**(C, D)** The scores of 13 immune-related functions were determined by ssGSEA analysis based on risk groups in the TCGA cohort and the ICGC cohort. **(E)** The fraction of different immune infiltration cells in low- and high-risk groups. **(F)** Relevance of risk scores to RNAs, DNAs, Immune, and Stromal Score. **(G)** Immune checkpoints and gene expression in high- and low-risk groups. **(H)** The association between immune subtypes and risk groups in HCC patients. *P<0.05, **P<0.01, ***P<0.001, ns, not statistically.

To evaluate the immune activity of the mitochondria-related gene signature, we first investigated the immune cells and immune functions using ssGSEA in low- and high-risk groups. As shown in **Figures 4A** **, B**, the nine immune cells and seven immune functions were statistical significance between two groups in the TCGA cohort. However, in the ICGC cohort, only the two (NK_cells and Th2_cells) immune cells were associated with risk groups (**Figures 4C** **, D** **)**. Compared with the high-risk group, the activity of the Type_II IFN response was upregulated in the low-risk groups.

Then, according to 22 types of immune cells, we draw a detailed immune cell compositions in HCC. The CD8+ T cells, M0 macrophages, and CD4+ T cells naive were the prime cell compositions in the high-risk group, whereas plasma cell in the low-risk group (**Figure 4E** **)**. We further analyzed tumor stemness, immune, and stromal cell scores to illuminate the role of the mitochondria-related gene signature in the tumor microenvironment. The results indicated that risk score was positively correlated with RNA stemness score (R = 0.28, P = 1.2e−07), while stromal scores were negatively correlated (R = −0.160.28, P = 0.003) (**Figure 4F** **)**. Moreover, the immune checkpoint analysis suggested that almost all the relevant genes in high-risk groups were significantly higher than those in low-risk groups (**Figure 4G** **)**.

Subsequently, we explored the relationship between risk groups and partial immune checkpoints (CTLA4, CXCR2, GPC3, HAVCR2, PDCD1, and PDL1). As shown in **Supplementary Figure 2**, the partial immune checkpoints except PDL1 were elevated significantly in the high-risk group. The expression of these immune checkpoints was positively correlated with the risk score. In addition, based on TCGA, the HCC patients were classified into four immune subtypes. The C3 and C4 subtypes are the prime immune subtypes, with a proportion of 82%. Most patients in the low-risk group are C3 (n = 80, 45%), C4 (n = 82, 46%) subtypes, while C4 (n = 77, 44%) is less common (**Figure 4H** **)**.

### Consensus clustering analysis of the prognosis and immunity based on mitochondria-related gene signature

**Figure 5:**
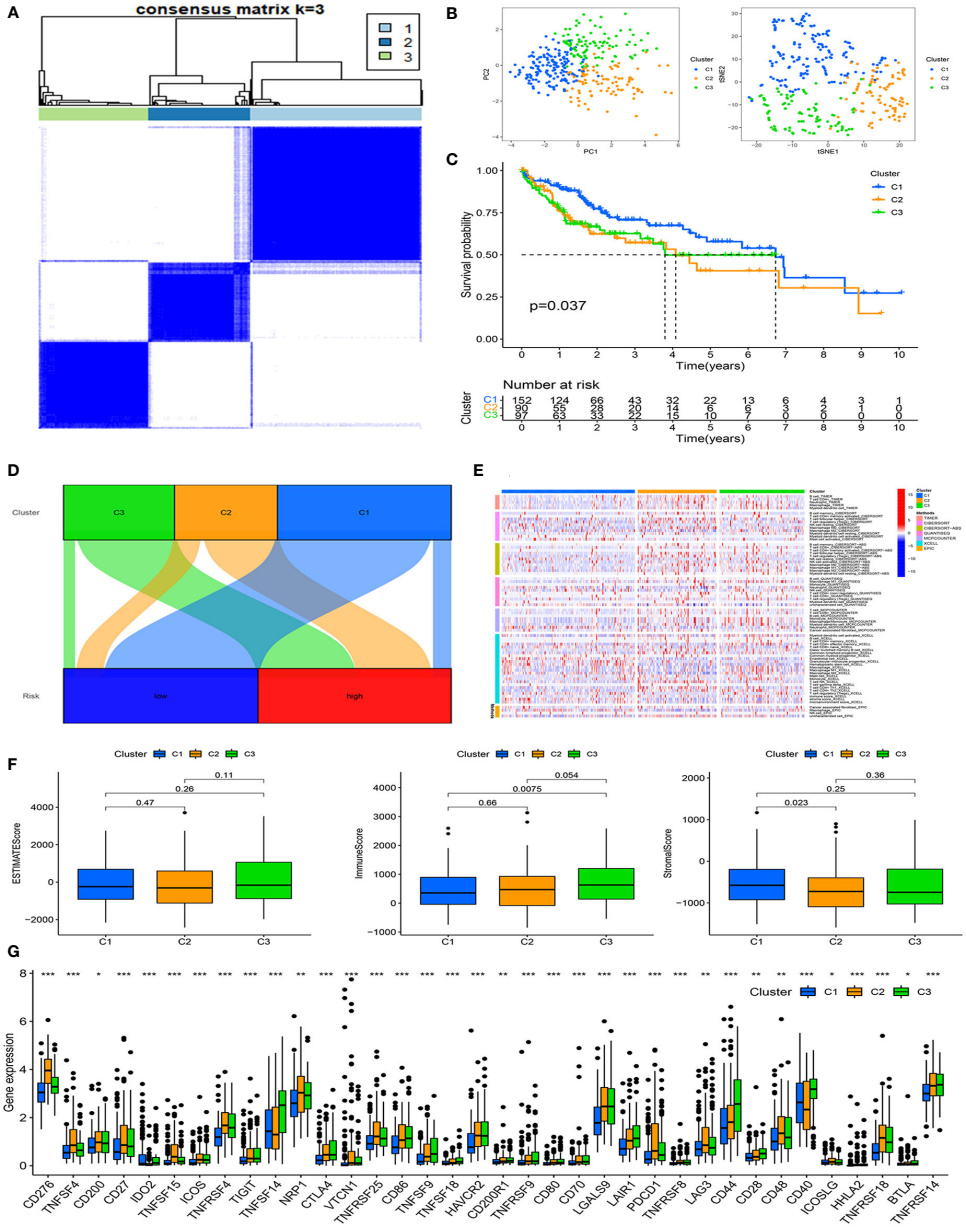
Consensus clustering analysis of the prognosis and immunity based on the mitochondria-related gene signature. **(A)** Identification of the optimal consensus matrix value. **(B)** The distribution of consensus clusters by PCA and tSNE analysis. **(C)** The prognosis of clusters C1, C2, and C3 by survival analysis. **(D)** The relationship between clusters and risk groups by alluvial diagram. **(E)** Analysis of the association between clusters and immune cells in different algorithms. **(F)** Analysis of the connection between clusters and ESTIMATE Score, Immune Score, and Stromal Score. **(G)** Analysis of the concern between clusters and immune checkpoints. *P<0.05, **P<0.01, ***P<0.001.

With the expression profiles of six mitochondria-related genes, we regrouped HCC patients into three clusters (cluster 1, cluster 2, and cluster 3) by consensus clustering analysis (**Figure 5A**). We verified the distributions of the above three clusters using PCA and tSNE analysis, implying that these clusters were distinguished (**Figure 5B**). Next, Kaplan–Meier analysis suggested that the survival of cluster 1 was longer than that of other clusters (**Figure 5C** **)**. Moreover, we visualized the relationship between clusters and risk groups in an alluvial diagram (**Figure 5D** **)**. Cluster 1 was primarily associated with the low-risk group, while clusters 2 and 3 were related to the high-risk group.

Concerning immunity, clusters 2 and 3 were closely associated with immune cells infiltrating (**Figure 5E** **)**. The ESTIMATE score indicated there were no significant differences among the three clusters (**Figure 5F** **)**. Cluster 1 had a higher stromal score compared to cluster 2. Cluster 3 had a higher immune score, signifying a more diverse tumor microenvironment than cluster 1. Besides, the expression of immune checkpoints was more abundant in clusters 2 and 3, indicating that they were susceptible to immunotherapy (**Figure 5G** **)**.

### Functional enrichment analysis based on risk groups

**Figure 6:**
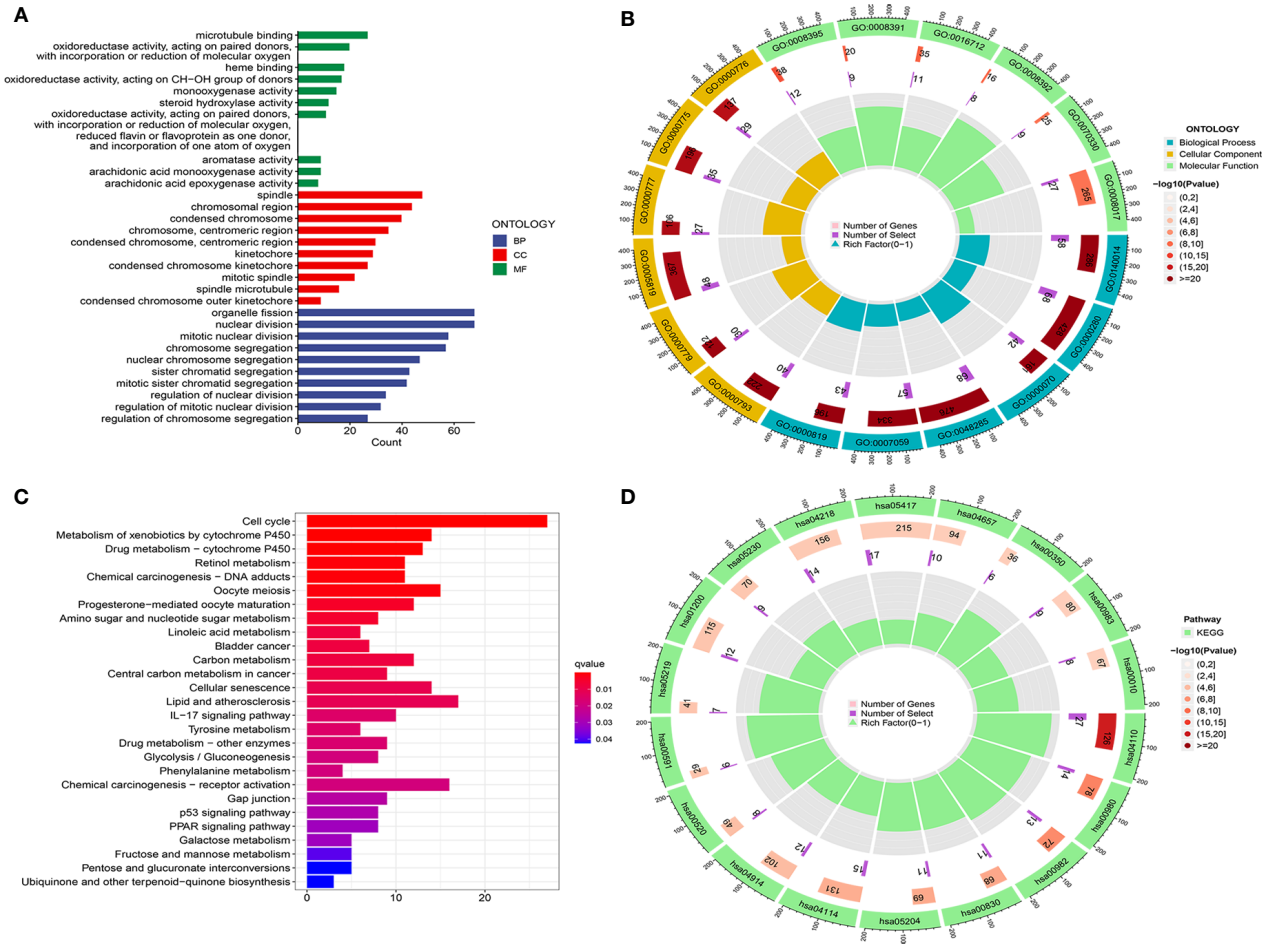
Analysis of functional enrichment. **(A, B)** GO analysis using a bar diagram and chordal graph. **(C, D)** KEGG analysis using a bar diagram and chordal graph.

To explore potential biological functions, we carried out GO and KEGG analyses in low- and high-risk groups. In **Figures 6A** **, B**, we found that the richest biological processes were related to organelle fission, nuclear division, mitotic nuclear division, and chromosome segregation. The first three cellular components were the spindle, the chromosome region, and the condensed chromosome. Moreover, in terms of molecular functions, microtubule binding, oxidoreductase activity (acting on paired donors with incorporation or reduction of molecular oxygen and acting on the CH-OH group of donors), and heme binding. In addition, the KEGG analysis showed that the risk groups jointly participated in the cell cycle, metabolism of xenobiotics, drug metabolism, oocyte meiosis, lipid atherosclerosis, and retinol metabolism. The above results concluded that its functions were closely associated with oxidation, metabolism, and the cell cycle (**Figures 6C** **, D** **)**.

### Analysis of drug sensitivity based on risk groups and clusters

To further improve the therapeutic effect on HCC patients, we investigated the relationship between risk groups and clusters and drug sensitivity. We identified significant differences in the IC50 of drugs between low- and high-risk groups, including Imatinib, Tipifarnib, Epothilone, Doxorubicin, and Bosutinib (**Supplementary Figures 3A–E**). Moreover, the results of IC50 associated with various drugs among clusters were consistent with those in the risk groups (**Supplementary Figures 3F–K** **)**. Therefore, considering the mitochondria-related model, the prognosis of HCC patients is expected to be greatly improved in the future.

### Experimental verification of the mitochondria-related gene expression by immunohistochemistry and qRT-PCR analysis

**Figure 7:**
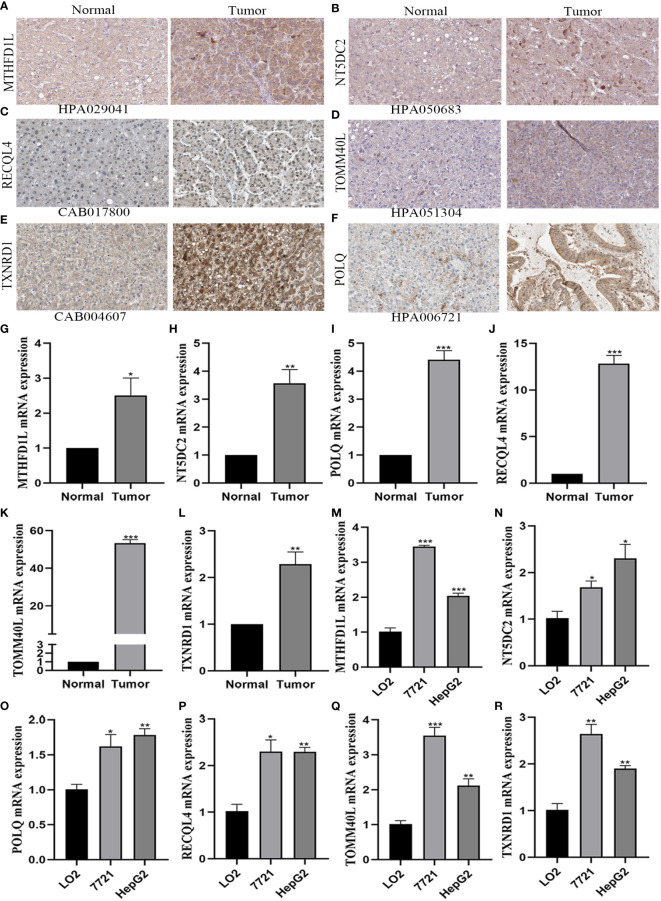
Verification of the expression of six mitochondrial-related genes by immunohistochemistry and qRT-PCR analysis. **(A–F)** The protein expression of six mitochondrial-related genes in HCC and adjacent normal tissues by immunohistochemistry. **(A)** MTHFD1L. **(B)** NT5DC2. **(C)** RECQL4. **(D)** TOMM40L. **(E)** TXNRD1. **(F)** POLQ. **(G–R)** The mRNA expression of six mitochondrial-related genes in HCC tissues and cell lines by qRT-PCR analysis. **(G, M)** MTHFD1L. **(H,N)** NT5DC2. **(I, Q)** POLQ. **(J, P)** RECQL4. **(K, Q)** TOMM40L. **(L, R)** TXNRD1. *P<0.05, **P<0.01, ***P<0.001.

The protein expression of 6-gene was analyzed by immunohistochemistry in the HPA database. In **Figures 7A–F**, we found that the 6-gene protein were positively expression in HCC tissues compared to normal tissues. Moreover, MTHFD1L, NT5DC2, POLQ, TOMM40L, and TXNRD1 were in cytoplasmic or membranous, while RECQL4 was in nuclear. Based on the above results, we further investigated the expression of six mitochondria-related genes by RT-qPCR analysis. Compared with normal tissues, MTHFD1L, NT5DC2, POLQ, RECQL4, TOMM40L, and TXNRD1 expression were upregulated in the 10 pairs of HCC tissues (**Figures 7G** **–L** **)**. In terms of HCC cell lines (7721 and HepG2), these six genes were identified with the results of HCC tissues. So, the mitochondria-related genes could be a potential biomarker for HCC (**Figures 7M** **–R** **)**.

## Discussion

Mitochondrial homeostasis is maintained by the nuclear genome and the genome encoding mitochondrial proteins (18). Mitochondrial dysfunction gives rise to decreased oxidative phosphorylation and increased resistance to glycolysis and radiotherapy, resulting in metabolic reprogramming and lipid oxidative damage in cancers (19–21). Recently, the imbalance of mitochondrial function has been closely related to the occurrence of various tumors (22). For example, the upregulated DRP1 or downregulated MFN1 gene contributed to HCC survival by increasing mitochondrial fission through ROS-mediated AKT activation (23). Thus, it is very necessary that we distinguish a persuasive signature based on mitochondria-related genes to definite the comprehensive roles in patients with HCC.

In this study, combined with 32 DEGs of mitochondria-related genes and 106 prognostic genes, we identified eight intersection genes for follow-up studies in HCC and normal tumors. Then, the six mitochondria-related gene signature was constructed for better forecasting the prognosis and immunity in the TCGA cohort. From the results of the model presentation, patients in high-risk group had a shorter overall survival than those in low-risk group, indicating a notably poorer prognosis of HCC patients in these mitochondria-related genes. The ROC curve, nomogram, and calibration analysis exhibited an admirable clinical predictive performance of the model. Subsequently, the risk score was associated with clinicopathological characteristics, and proving to be an independent prognostic factor in patients with HCC. Following that, we further determined the differences in immunity and drug sensitivity between low- and high-risk groups. To our satisfaction, the above results in the TCGA cohort were identified with the ICGC cohort. Besides, based on risk groups, we regrouped into three clusters for evaluating the value of mitochondria-related genes one more by the consensus clustering analysis. Moreover, the functional enrichment analysis revealed that the mitochondria-related genes played vital roles in chromosome separation, oxidation, and metabolism between low- and high-risk groups. In addition, the relevant experiments were applied to verify the mRNA expression of six mitochondria-related genes in tissues and cell lines.

For these genes with an established signature (MTHFD1L, NT5DC2, POLQ, RECQL4, TOMM40L, and TXNRD1), we further investigated their potential functions and research advances. Metabolic disorders of folic acid had an influence on the methylation process, DNA synthesis, and repair and then led to different kinds of diseases (24). MTHFD1L, a mitochondrial protein with tetrahydrofolate synthase activity, not only plays an important role in supporting cancer growth but also increases susceptibility to targeted therapy (25). Recent studies indicated that knockdown of MTHFD1L may inhibit proliferation and induce apoptosis in tumor cells by regulating c-MYC and p53-related pathways (26–28). Genome-wide association studies have identified NT5DC2 as a neuropsychiatric disorder associated with abnormal dopamine activity (29). NT5DC2 can promote the tumorigenesis of glioma stem cell-like cells by inducing the expression of the FYN protooncogene and increase the proliferation of tumor cells in HCC by stabilizing the epidermal growth factor receptor (30, 31). Moreover, NT5DC2 expression was related to prognosis in HCC and may be a promising biomarker for HCC stratification (32). POLQ is a DNA polymerase involved in processes such as transcriptional DNA synthesis and double-strand break repair, which are associated with poor prognosis and a characteristic mutation signature (33, 34). POLQ inhibitors are a promising cancer treatment strategy in which cancer cells use DNA repair gene mutations to reconnect their DNA repair network to compensate for survival (35). RECQL4, a member of the RECQ helicase family, plays a key role in the maintenance and stability of the nuclear and mitochondrial genomes (36). Recent studies have shown that RECQL4 is overexpressed in most cancers and is associated with clinical outcomes (37, 38). TOMM40L is a potential channel-forming protein whose main function is to import protein precursors into mitochondria (39). Although polymorphisms encoding TOMM40 have been shown to be associated with an increased risk of late-onset Alzheimer’s disease (40), further studies are needed to elucidate the function and mechanism of TOMM40L in the future. The TrxR/Trx system is one of the cores of the cellular REDOX regulatory network (41). TXNRD1, a form of TXN reductase, is upregulated in various cancers, including HCC, and is considered an adverse prognostic factor (42). Enhanced TXNRD1 expression was associated with tumor progression and metastasis and conferred chemotherapy resistance (43). Therefore, the signature may serve as a critical model for evaluating the prognosis of patients with HCC.

Next, we investigated the immune status and tumor microenvironment to identify the immune activity of the mitochondria-related gene signature. In the TCGA cohort, there were nine immune cells and seven immune functions with statistical significance in both low- and high-risk groups, but only NK_cells and Th2_cells were associated with risk groups in the ICGC cohort. We also found the upregulated Type_II IFN response in the low-risk group which means the immune surveillance mechanism is still alive and natural killer T cells remain producing IFN-γ and activating NK cells to inhibit tumor growth in this group (44). Then, according to 22 types of immune cells, we draw a HCC component immune cell picture, and tumor stemness, immune, and stromal cell scores show that the risk scores were positively correlated with the RNA stemness score, which was higher in the high-risk group, and negatively with the stromal cell scores, which were higher in the low-risk group. The immune checkpoints analysis outcome also complies with the characteristics of high-risk group with HCC more relevant genes. Moreover, referring to previous reports, the molecular subtypes, also called clusters, are associated with tumor immune suppression and microenvironments (45). Different subtypes have different immune and TME scores, leading to different immunotherapy responses (46). According to our results, the patients with HCC were classified into four different immune subtypes. The C3 and C4 subtypes were the prime immune subtypes, but the C1 and C2 subtypes had a bad prognosis in HCC. Therefore, the relevant immune value of the mitochondria-related gene signature was sufficient.

To date, this is the first study to comprehensively identify and verify the prognostic and immune value of a mitochondria-related gene signature in HCC patients, which shows a favorable model for clinical application. However, some limitations need to be addressed in this study. Firstly, we may have ruled out other genes unrelated to mitochondria that are associated with the prognosis of HCC. Secondly, although we validated the expression of prognostic genes, basic research is needed to explore the underlying functions. Besides, the established signature is based on retrospective data, which requires further validation of prospective data from multi-center research.

## Conclusion

Our study successfully constructed and fully validated the prognostic signature based on six mitochondria-related genes, and not just presented a preferable superiority in distinguishing immune therapy response, but also may possess vital guidance value for clinical diagnosis and treatment. Future studies should further illuminate the potential mechanisms by which the 6-gene signature regulates the immune microenvironment and offer a fundamental theory for HCC drug targets.

## Data availability statement

The original contributions presented in the study are included in the article/**Supplementary Material**. Further inquiries can be directed to the corresponding authors.

## Ethics statement

The collection of human tissue samples for this study was approved and authorized by the ethics committee of Fuyang Hospital of Anhui Medical University.

## Author contributions

YS, GH, JZ, and FJ contributed to the conceptualization, performed the data, and wrote the manuscript. QX and ZP also wrote the draft and review version. SL, HJ, and HF performed the experiments. YZ and LL provided constructive advises in whole process. YZ contributed to the funding. All authors listed have made a substantial, direct, and intellectual contribution to the work and approved it for publication.

## Funding

This study was supported by the National Natural Science Foundation of China (62141109), the Foreword Leading Technology Fundamental Research Project of Jiangsu (BK20212012), the Jiangsu Province Social Development Project (BE2022812), and the Research Fund of Anhui Medical University (2020xkj057).

## Conflict of interest

The authors declare that the research was conducted in the absence of any commercial or financial relationships that could be construed as a potential conflict of interest.

## Publisher’s note

All claims expressed in this article are solely those of the authors and do not necessarily represent those of their affiliated organizations, or those of the publisher, the editors and the reviewers. Any product that may be evaluated in this article, or claim that may be made by its manufacturer, is not guaranteed or endorsed by the publisher.

## Supplementary material

Supplementary Figure 1 The prognosis of 6 mitochondria-related genes were investigated by survival analysis with optimal cut-off expression. **(A)** MTHFD1L. **(B)** NT5DC2. **(C)** POLQ. **(D)** RECQL4. **(E)** TOMM40L. **F)** TXNRD1. *P* < 0.05.

Supplementary Figure 2 Analysis of the relationship between immune checkpoints and risk groups, risk score. **(A)** CTLA4. **B)** CXCR2. **(C)** GPC3. **(D)** HAVCR2. **(E)** PDCD1. **(F)** CD274. *P* < 0.05.

Supplementary Figure 3 Analysis of drug sensitivity based on risk groups and clusters. **(A, F)** Imatinib. **(B, H)** Tipifarnib. **(C, I)** Epothilone. **(D, J)** Doxorubicin. **(E, K)** Bosutinib. *P* < 0.05.
