# Supplementary figures and images for: Deciphering a mitochondria-related signature to supervise prognosis and immunotherapy in hepatocellular carcinoma
Source: Front Immunol. 2022 Dec 2;13:1070593. doi: 10.3389/fimmu.2022.1070593 (PMC9761315; doi:10.3389/fimmu.2022.1070593)

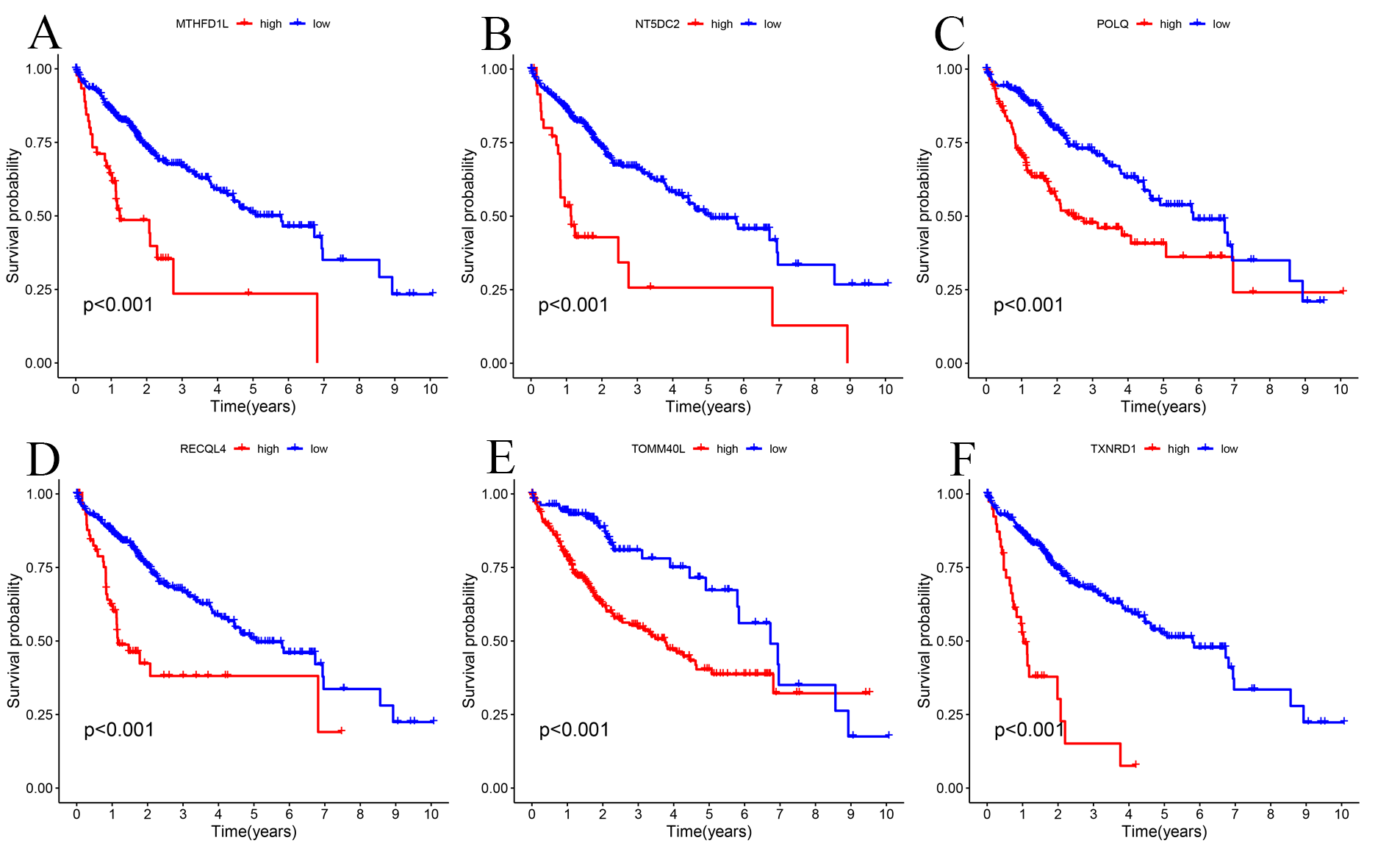

Supplement: Supplementary Figure 1 — The prognosis of 6 mitochondria-related genes were investigated by survival analysis with optimal cut-off expression. (A) MTHFD1L. (B) NT5DC2. (C) POLQ. (D) RECQL4. (E) TOMM40L. F) TXNRD1. P < 0.05. [file Image_1.tif]

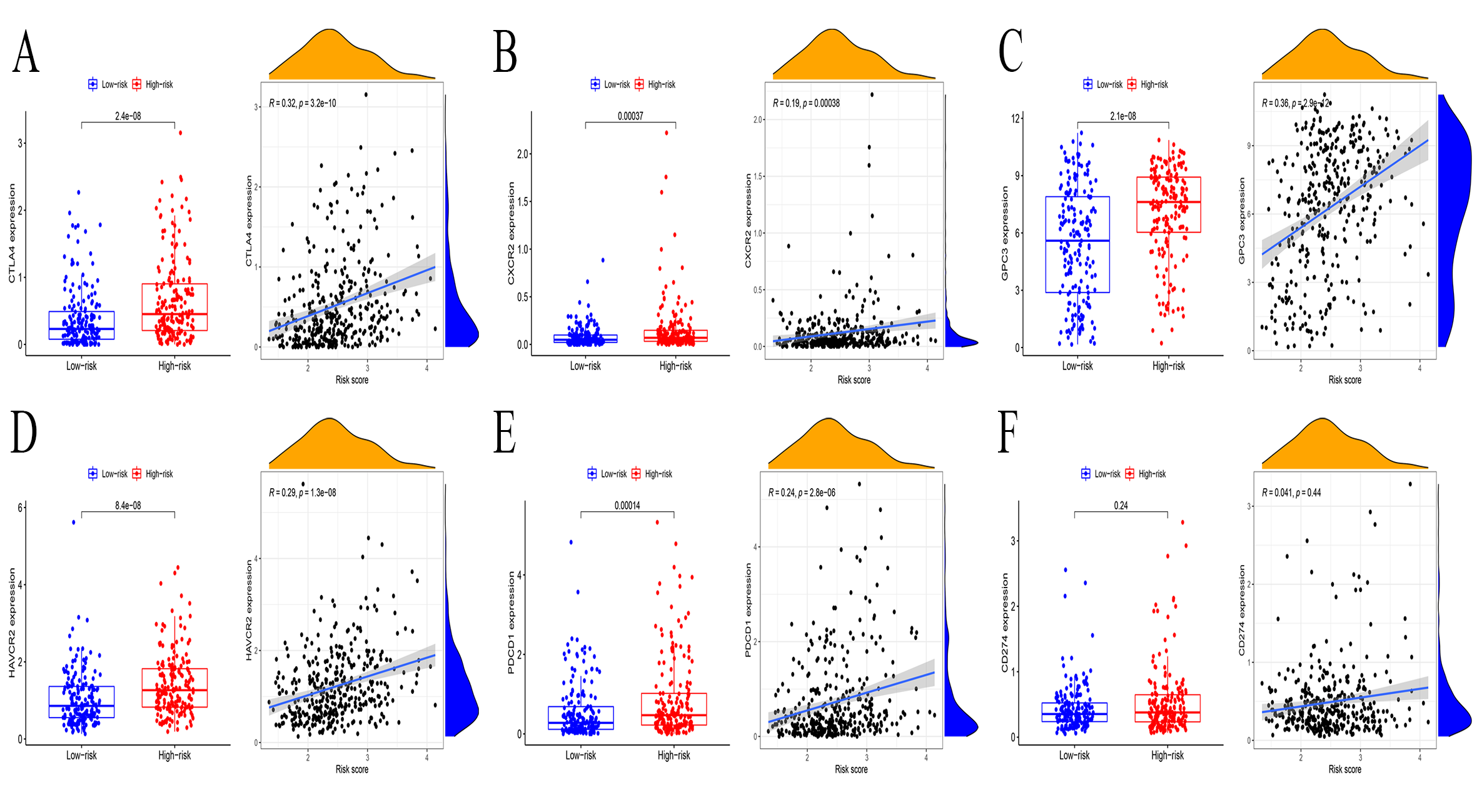

Supplement: Supplementary Figure 2 — Analysis of the relationship between immune checkpoints and risk groups, risk score. (A) CTLA4. B) CXCR2. (C) GPC3. (D) HAVCR2. (E) PDCD1. (F) CD274. P < 0.05. [file Image_2.tif]

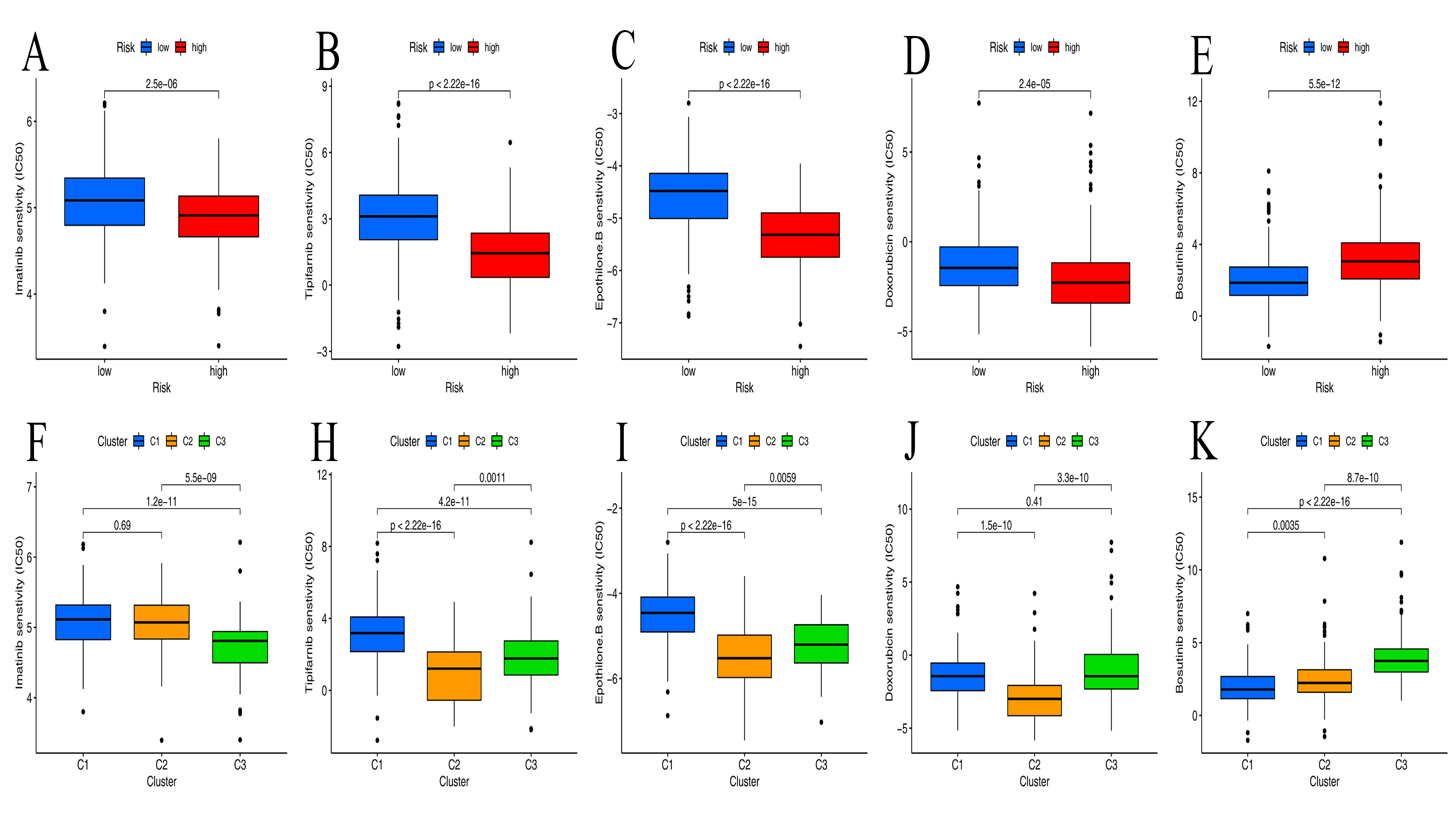

Supplement: Supplementary Figure 3 — Analysis of drug sensitivity based on risk groups and clusters. (A, F) Imatinib. (B, H) Tipifarnib. (C, I) Epothilone. (D, J) Doxorubicin. (E, K) Bosutinib. P < 0.05. [file Image_3.tif]
